# Mindfulness-based intervention for hypertension patients with depression and/or anxiety in the community: a randomized controlled trial

**DOI:** 10.1186/s13063-024-08139-0

**Published:** 2024-05-02

**Authors:** Hailiang Zhang, Xiangrong Zhang, Xiaomei Jiang, Runjing Dai, Na Zhao, Weimin Pan, Jiaohong Guo, Jingchun Fan, Shisan Bao

**Affiliations:** 1 Center for Laboratory and Simulation Training, School of Public Health, Center for Evidence-Based Medicine, Gansu University of Chinese Medicine, Lanzhou, 730101 Gansu China; 2 Collaborative Innovation Center for Traditional Chinese Medicine Prevention and, Control of Environmental and Nutrition-Related Diseases in Northwest China, Lanzhou, 730101 Gansu China; 3 Department of Mental Health, Gansu Provincial Centre for Disease Control and Prevention, Lanzhou, 730030 Gansu China; 4 Department of Chinese Medicine, Center of Hekou Town, Xigu District, Lanzhou, 730094 Gansu China; 5 Department of Psychosomatic and Sleep Medicine, Gansu Gem Flower Hospital, Xigu District, Lanzhou, 730060 Gansu China; 6 Department of Hospital Infection-Control, Affiliated Hospital of Gansu University of Chinese Medicine, Lanzhou, 730020 Gansu China; 7 Department of Vasculo-Cardiology, Pingliang Second People’s Hospital, Kongtong District, Pingliang, 744000 Gansu China

**Keywords:** Hypertension, Anxiety, Depression, Mindfulness-based therapy, Blood pressure (BP)

## Abstract

**Objective:**

To evaluate mindfulness-based intervention for hypertension with depression and/or anxiety.

**Methods:**

10-week mindfulness-based intervention, including health education for hypertension, exclusively for the control group, was administered to the intervention group to assist sixty hypertension patients with depression/anxiety. Among them, the intervention group comprised 8 men and 22 women, with a mean age of 60.02 years and a mean duration of hypertension of 6.29 years. The control group consisted of 14 men and 16 women with a mean age of 57.68 years and a mean duration of hypertension of 6.32 years. The severity of depressive and/or anxiety symptoms was assessed using the 9-item Patient Health Questionnaire (PHQ-9) and the 7-item Generalized Anxiety Disorder scale (GAD-7), along with blood pressure (BP) measurements taken twice daily. The study utilized a self-made self-efficacy scale and awareness of physical and mental health to evaluate mental health and state.

**Results:**

The depression PHQ-9 or GAD-7 scores reduced by 21.1% or 17.8% in the mindfulness-based intervention group, compared to the control (Z = -2.040, *P* = 0.041) post 10-week period, suggesting significant reduction in anxiety/stress. These results were consistent with a reduction in systolic BP of 12.24 mm Hg (t = 6.041, *P* = 0.000). The self-efficacy score of the mindfulness intervention group significantly improved compared to the control (t = 7.818, *P* < 0.001), while the awareness of physical and mental health in the mindfulness intervention group significantly improved compared to the control (χ2 = 5.781, *P* = 0.016).

**Conclusion:**

Mindfulness-based, short-term focused interventions provide modest relief for depression and/or anxiety and are effective in lowering blood pressure and improving self-efficacy scores.

**Trial registration:**

Chinese Clinical Trial Registry, ChiCTR1900028258. Registered 16 December 2019, https://www.chictr.org.cn/showproj.html?proj=43627.

**Supplementary Information:**

The online version contains supplementary material available at 10.1186/s13063-024-08139-0.

## Introduction

Hypertension, a common chronic disease with a high prevalence (27.9%) among Chinese adults [1], usually requires lifelong management of blood pressure (BP) with medications [2]. A high number of hypertensive patients also experience co-existing depression and/or anxiety [3]. Additionally, a significant portion of these hypertensive patients are non-compliant with medical practitioners' instructions, resulting in serious complications and compromised quality of life [4]. Therefore, managing depression and/or anxiety is equally as important as treating hypertension.

There has been documented a close association between hypertension and depression or anxiety, which are independent risk factors for hypertension [5]. Stress relief is therefore an ideal approach in the ongoing management of hypertension [6]. Psychotherapy for hypertensive patients with depression and/or anxiety improves both psychological symptoms and blood pressure [7]. Studies on psychotherapy typically focus on hypertensive patients in hospitals with severe depression and/or anxiety [8]. Only a few reports review the management of these hypertensive patients with depression and/or anxiety in the community [9]. Despite the lack of studies, it is acknowledged that the majority of hypertensive patients with depression and/or anxiety, except for extremely severe cases, are managed at the community level in China [10]. Hypertensive patients are usually managed with a cocktail of pharmacological agents to control blood pressure; however, the outcomes may not be satisfactory [11], partly due to medical non-compliance.

Mindfulness has been used for self-regulation of chronic pain since 1985 and has shown short- and long-term improvements [12]. Hofmann and Gómez describe mindfulness-based stress reduction and mindfulness-based cognitive therapy as “a combination of the essence of Eastern mindfulness-based practices with Western cognitive-behavioral practice” [13]. The three components of mindfulness-based therapy include avoiding judgment, enhancing awareness, and focusing on the present moment. Paying attention to the present helps individuals to process their cognitive, physiological, and behavioral activities. Such modification occurs through the regular practice of mindfulness-based activities, which makes individuals become aware not only of their daily activities but also of the automatic function of their mind in the past and future [14]. Interestingly, functional training (TF), which has similar effects to mindfulness, can also reduce anxiety levels and improve poor lifestyle habits [15, 16].

The standard practice for mindfulness-based therapy among hypertensive patients is a group-based program, including daily group practice and quiet individual practice to harmonize their thoughts, emotions, feelings, and behaviours [17]. Global health has been developed over the last decade to improve health for people in need at the global level, including mental health [18]. For example, mindfulness-based therapy has become a standardized treatment with rather impressive outcomes and may be a potential non-pharmacological alternative approach to managing hypertension in the community [19–21]. Mindfulness-based therapy provides hypertensive patients with depression and/or anxiety a tool to deal with their negative feelings and physical symptoms through a relaxing approach, perhaps by increasing their threshold or reducing their sensitivity to problems [22].

We aimed to evaluate whether mindfulness-based intervention can reduce psychological stress and, subsequently, hypertension among hypertensive patients with depression and/or anxiety.

## Methods

### Participants

Using the Patient Health Questionnaire Depression Scale (PHQ-9) [23], the sample size was calculated based on the baseline or final score, which were set as 4.58 or 9.27, respectively, as the outcome indicators.

The specific calculation formula used for the current study is as follows [24]:

$$N=2({t}_{\alpha }+{t}_{\beta }{)}^{2}{S}^{2}/^{2}$$

$${\text{Among}}, {t}_{\alpha }=1.96, {t}_{\beta }=1.64, S=4.77, =4.69$$

Therefore, N≈26.81, indicating that 27 participants in each of the intervention and control groups would need to be included, totalling 54 participants. Initially, 80 volunteers were recruited, but only 60 participants were involved in the trial.

We recruited 60 participants with hypertension (defined as a mean awake ambulatory systolic or diastolic BP ≥ 140/90 mm Hg) and depression (PHQ-9 evaluation score ≥ 5) between August 2020 and October 2020. They were recruited from referrals by physicians and posters in the local community surrounding Gem Flower Hospital, Lanzhou, Gansu Province, China. Among these participants, depression and anxiety levels were measured using the PHQ-9 (Patient Health Questionnaire 9-items) and GAD-7 scale (Generalized Anxiety Disorder scale-7 items) to assess comorbid depression and/or anxiety. The study officially commenced on November 6th, 2020, and concluded on January 2nd, 2021, comprising a total of 77 days of continuous intervention.

The PHQ-9 and GAD-7 scales are relatively favoured for depression screening among individuals with depression and/or anxiety due to their simplicity and time efficiency. We selected the PHQ-9 depression and GAD-7 anxiety scales because they are believed to possess high reliability and validity and are widely used internationally as generic questionnaires for depression [25] and anxiety [26]. Therefore, in the current study, we chose the PHQ-9 and GAD-7 scales to evaluate depression and/or anxiety in participants, while participants' mental health was also assessed through a simple self-efficacy scale. This self-efficacy scale, which has been widely used over the last few decades [27], was adopted for the current study. The reliability and validity of the self-efficacy scale are as follows: the results for construct, convergent, and discriminant validity of the scale are good. Exploratory factor analysis produced eigenvalues between 2.27 and 3.28, with the factors explaining a total cumulative variance of 91.1%. Overall, the reliability and consistency of the self-efficacy scale are good [28].

The self-efficacy scale primarily assesses participants' subjectivity, initiative, and social and family support, likely reflecting their mental health and the importance of self-management for disease recovery [29].

Participants' BP was measured twice daily at 10 am and 2 pm using a mercury sphygmomanometer in a relatively quiet state, with the average BP used for the current study.

The effect of mindfulness-based meditation on hypertensive patients in the community in controlling BP following 10 weeks of intervention was evaluated, with a particular focus on whether such intervention could improve negative emotions in hypertensive patients. The current study adheres to the Declaration of Helsinki and has been approved by the Human Ethics Committee of Gansu Gem Flower Hospital (No. 201902)."

### Inclusion criteria

1. Hypertensive patients (systolic BP > 140 mmHg and/or diastolic BP > 90 mmHg).
2. Depression scale (PHQ-9) evaluation score ≥ 5 points.
3. Anxiety scale (GAD-7) evaluation score ≥ 5 points.
4. Aged 18-69 years.
5. Ability to understand the questionnaires without significant barriers to daily communication.
6. Planning to stay at the current residence for the next 6 months.

### Exclusion criteria

1. Patients with severe myocardial infarction, cerebral infarction, cirrhosis, or uraemia.
2. Physical incapability to participate in the project.
3. Severe vision and hearing impairments.
4. Taking antipsychotics, anti-anxiety, and depression medications within the past 3 months.
5. Substance abuse.
6. Alzheimer's disease.

### Study design and intervention

This study was a prospective, double-blind research (where treatment assignments were concealed from those who organize and analyze the data) randomized trial. A random number table was used to assign these 60 participants into the mindfulness-based intervention and control groups (*n* = 30 each).

The recruited patients had comorbid hypertension and anxiety/generalized anxiety disorders. The control cohort received a 2-h lecture weekly on general information about hypertension, provided by specialized cardiologists. In addition to the lectures, the intervention group also received weekly education on mindfulness-based therapy from specialized psychologists in hypertension mindfulness, along with Tai Chi sessions accompanied by relaxing background music and post-session consolidation. The specific teaching contents of the mindfulness therapy and hypertension health education are presented in Table 1. The primary outcomes (depression and anxiety scores) were assessed between the initial baseline and 10 weeks after with or without mindfulness-based intervention. Secondary outcomes, such as systolic or diastolic blood pressure (BP) and self-efficacy scores, were also assessed between the initial baseline and 10 weeks of intervention for all participants. This data could provide objective value for evaluating the severity of stress and/or depression [30].

**Table 1:** Details of each regimen for the two groups

Intervention content		Group	
		Intervention group	Control group
Mindfulness therapy	Relaxation training	✓	
	Ways to stay happy and seek social support	✓	
	Measurement of BP	✓	
	Negative thinking recognition	✓	
	Cognitive behaviour improvementis	✓	
	Broaden mental flexibility	✓	
	Mindfulness exercises	✓	
	Communication skills	✓	
HTN health education	Hypertension knowledge	✓	✓
	Influencing factors of hypertension	✓	✓
	Self-test BP	✓	✓
	The importance of controlling BP	✓	✓
	Proper use of anti-hypertensive drugs	✓	✓
	Management of hypertension	✓	✓
	Reasonable diet	✓	✓
	Good living customs	✓	✓
	Fitness campaign	✓	✓
	Conclusion and prospect	✓	✓

In this randomized controlled trial, we did not establish a special project management team; however, in actual practice, our staff assumed the responsibility of project management. Our staff held meetings during the participant recruitment period, the trial period (the fifth week of the trial), and the data collection period (weekly) to review the RCT. The main contents of the review mainly included assessing whether the participants met the inclusion and exclusion criteria, reviewing the duration and content of the intervention, ensuring that the outcome indicators were measured, and checking for errors or omissions in the collected data.

### Measurement scales and scoring criteria

The initial baseline data in this study included age, education level, marital status, BMI, duration of hypertension, presence of diabetes mellitus, systolic blood pressure, diastolic blood pressure, depression score, anxiety score, and self-efficacy score.

The PHQ-9 is a brief and self-explanatory questionnaire, serving as an efficient self-assessment tool for depression based on the American Diagnostic and Statistical Manual of Mental Disorders (DSM IV) [31]. It comprises 9 items, each with four options: "completely impossible," "several days," " > 1 week," and " ~ every day," with corresponding scores of 0, 1, 2, and 3 points, respectively. The highest combined score is 27 points, reflecting the severity of depression. The PHQ-9 scoring system has been reported as follows: 0–4 for normal, 5–9 for mild, 10–19 for moderate, and 20–27 for severe depression [32].

The GAD-7, primarily applied to patients with chronic diseases or the elderly population, is considered to possess reliability and validity [33]. It was utilized to validate the PHQ-9 system. The GAD-7 scale consists of 7 items, with the highest score being 21 points. Scores of 0–4 indicate normal anxiety, 5–9 indicate mild anxiety, 10–14 indicate moderate anxiety, and 15–21 prompt for severe anxiety [34].

In addition, the self-efficacy scale consists of 6 items, with scores ranging from 0 to 10, measuring perceived confidence in managing one's health. A higher score indicates a healthier psychological state.

### Statistics

Statistical analysis was performed using SPSS 25.0 software (International Business Machines Corporation). Measurement data that followed a normal distribution are presented as mean ± SD and were compared using Student’s t-test. For data that did not follow a normal distribution, median and interquartile range [M (IQR)] were used, and comparison was made using the Wilcoxon rank sum test. Intent-to-treat (ITT) analysis and power analysis were conducted to compare the results between the two groups. A significance level of α = 0.05 was used for hypothesis testing.

### Comparison of Intention to treat between two groups

To avoid selection bias and maintain comparability between the two groups, the study conducted an intention-to-treat (ITT) analysis using the PHQ-9 scale score as an outcome measure. Through ITT and power analysis, significant differences were observed between the two groups (χ2 = 29.433, *P* < 0.0001, χ2 = 30.583, *P* < 0.0001) (Table 2).

**Table 2:** Comparison of Intention to treat and power analysis between two groups

Methods	Group	n	Depression relief	Depression not relief	Depression control rate (%)	χ^2^	*P*
ITT	Intervention	30	25	5	83.3	29.433	< 0.0001
	Control	30	4	26	6.7		
Power analysis	Intervention	26	23	3	88.5	30.583	< 0.0001
	Control	29	4	25	13.8		

## Results

### Participants flow

**Fig. 1:**
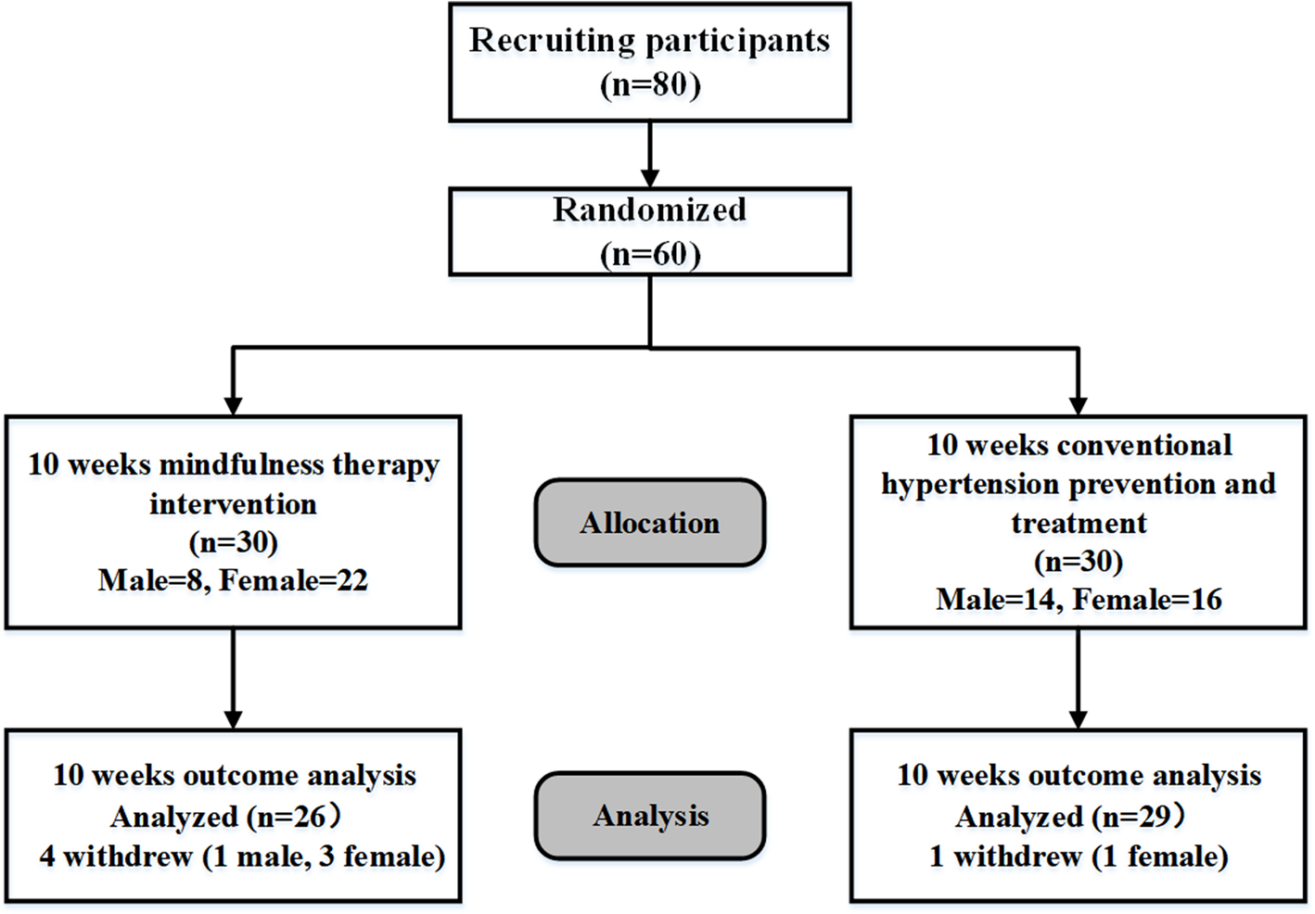
Subject intervention flowchart

It initially recruited 80 volunteers, of whom 11 were excluded for the reasons stated above. Among the remaining 69 participants, 8 declined to further participate, and 1 had a history of antidepressant medication. Thus, only 60 participants were finally recruited for the current study with informed consent (Fig. 1). These 60 participants were randomly divided into mindfulness-based intervention and control groups. At the end of the study, only 26 intervention participants completed the 10-week intervention. Another 4 participants were lost to follow-up, with 3 withdrawing early and 1 receiving antidepressant medication due to exacerbated depression. On the other hand, 29 participants in the control group completed routine hypertension prevention and treatment, with 1 participant quitting due to personal physical reasons.

### Baseline comparison between two groups

The initial baseline data in this study included age, education level, marital status, BMI, duration of hypertension, presence of diabetes mellitus, systolic blood pressure, diastolic blood pressure, depression score, anxiety score, and self-efficacy score. All participants with hypertension had not received antidepressant or anxiolytic therapy for more than 6 months prior to the study. The mean awake ambulatory blood pressure was 148.96 ± 17.82 / 85.24 ± 7.40 mmHg (systolic/diastolic) in the mindfulness-based intervention group, whereas it was 146.11 ± 10.80 / 82.68 ± 4.96 mmHg in the control group. There was no significant difference in baseline blood pressure between the intervention and control groups. Additionally, there was no significant difference in baseline anxiety and depression scores between the intervention and control groups, with scores of 9.27 ± 4.77 vs. 9.87 ± 4.62 (*P* = 0.609) for anxiety and 6.90 ± 2.56 vs. 6.83 ± 2.82 (*P* = 0.915) for depression (Table 3).

**Table 3:** Baseline comparison between intervention and control groups

Index	Intervention (*n* = 30)	Control (n = 30)	t/ χ^2^/Z	*P*
Age (Yr)	60.02 ± 5.23	57.68 ± 7.71	1.259	0.218
Male *vs* Female n (%)	8 (26.7) *vs* 22 (73.3)	14 (46.7) *vs* 16 (53.3)	2.584	0.108
Education				
≤ Primary school	11 (36.7)	15 (50)		
Middle school	13 (43.3)	7 (23.3)	5.400	0.067
≥ High school	6 (20.0)	8 (26.7)		
Marital status (%)				
Married	27 (90.0)	26 (86.7)		
Divorce	1 (3.3)	1 (3.3)	0.219	0.896
Windowed	2 (6.7)	3 (10.0)		
BMI, kg/m^2^	25.71 ± 2.50	24.70 ± 3.46	-1.230	0.219
Course of Hypertension (Yr)	6.29 ± 5.60	6.32 ± 4.45	0.371	0.711
DM *vs* Non-DM (%)	17 (56.6) *vs* 13 (43.4)	15 (50.0) *vs* 15 (50.0)	0.268	0.065
Systolic BP (mm Hg)	148.96 ± 17.82	150.17 ± 8.47	0.391	0.699
Diastolic BP (mm Hg)	85.17 ± 7.36	82.57 ± 2.97	-1.819	0.079
Depression score	9.27 ± 4.77	9.87 ± 4.62	-0.512	0.609
Anxiety score	6.90 ± 2.56	6.83 ± 2.82	-0.107	0.915
self-efficacy score	3.40 ± 0.724	3.77 ± 0.971	1.650	0.110

### Comparison of depression and anxiety scores and BP before and after intervention

**Fig. 2:**
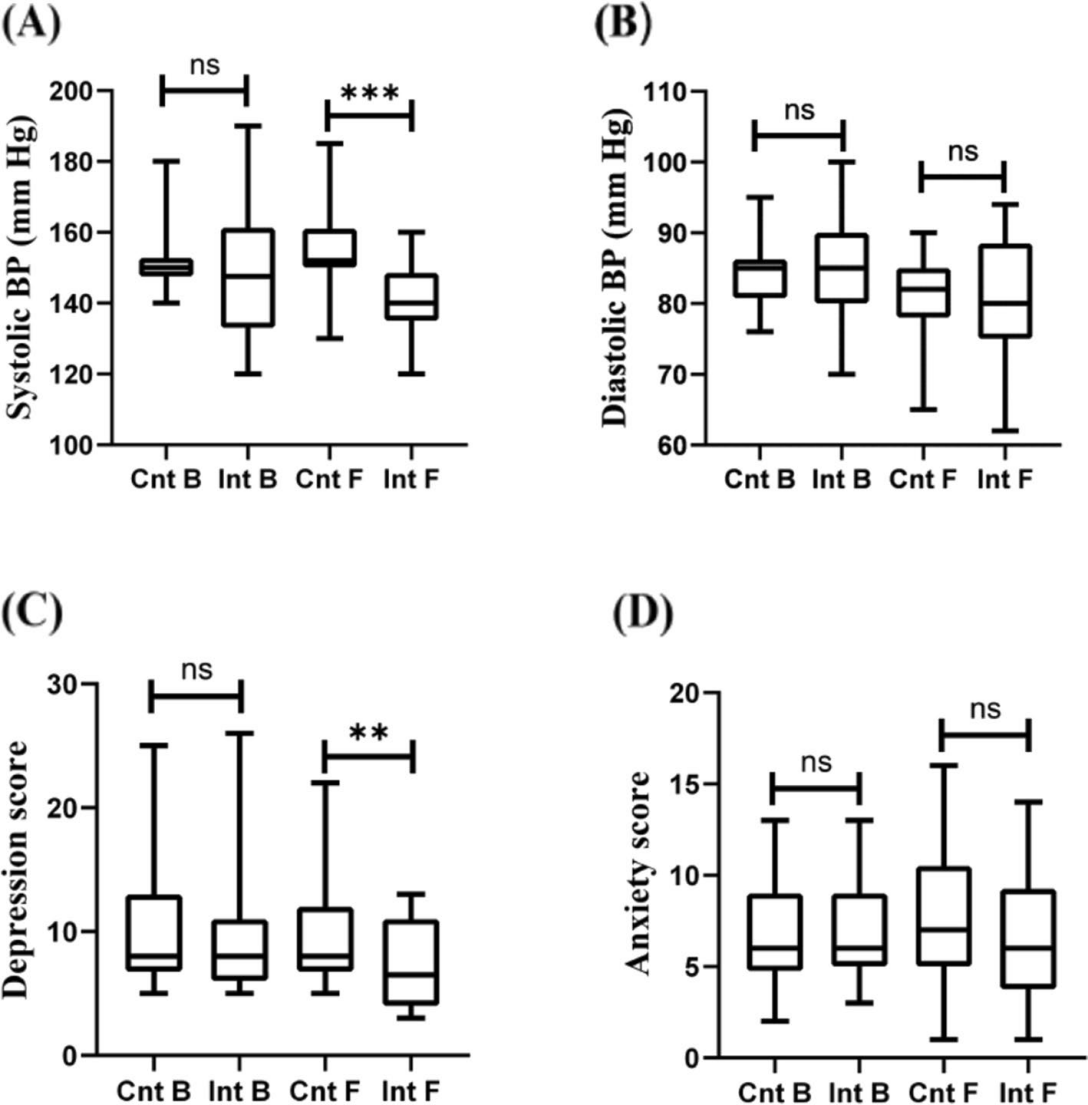
**A** Comparison of SBP before and after intervention in the two groups; **B** Comparison of DBP before and after intervention in the two groups; **C** Comparison of depression score before and after intervention in the two groups; **D **Comparison of anxiety score before and after intervention in the two groups. (ns:* P* > 0.05; **:*P* < 0.05; ***:*P* < 0.001)

There was no significant difference in depression (9.27 ± 4.77 vs. 9.87 ± 4.62) and anxiety scores (6.90 ± 2.56 vs. 6.83 ± 2.82) (*P* > 0.05), as well as systolic BP (148.96 ± 17.82 vs. 150.17 ± 8.47) and diastolic BP (85.17 ± 7.36 vs. 82.57 ± 2.97) (*P* > 0.05) between the intervention and control groups prior to the intervention. However, depression scores (7.42 ± 3.46 vs. 9.41 ± 3.98) (Z = -2.040, *P* = 0.041) and systolic BP (142.31 ± 9.33 vs. 154.55 ± 11.20) (t = 6.041, *P* < 0.0001) were significantly reduced from stage 2 to stage 1. Furthermore, there was no significant difference between these two groups in the reduction of anxiety score (6.35 ± 3.93 vs. 7.72 ± 3.93) (Z = -0.864, *P* = 0.388) and diastolic BP (80.62 ± 7.87 vs. 81.45 ± 5.56) (t = 0.253, *P* = 0.803) (Fig. 2).

### Comparison of self-efficacy score between two groups of patients before and after intervention

**Fig. 3:**
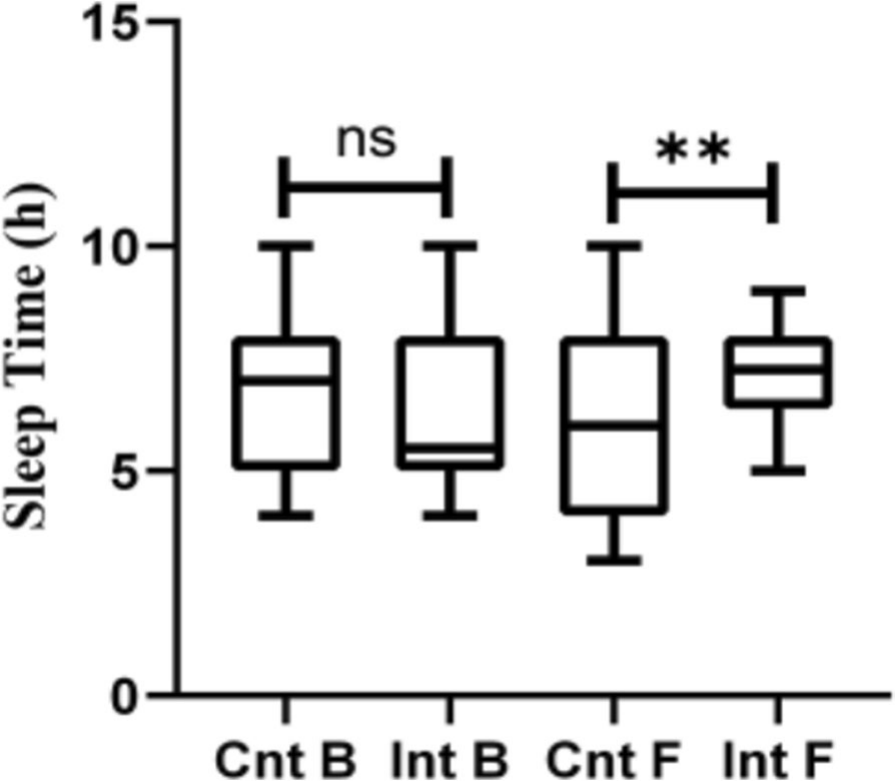
Comparison of self-efficacy score before and after intervention in the two groups

After the intervention, the self-efficacy score of participants in the intervention group was significantly higher than that of the control group (5.23 ± 1.45 vs. 3.86 ± 1.36) (t = 3.104, *P* = 0.002) (Fig. 3).

### Comparison of awareness rate of physical and mental health between two groups of patients before and after intervention

After the intervention, the awareness rate of physical and mental health among participants in the intervention group was significantly higher than that among the control group (χ2 = 5.781, *P* = 0.016) (Table 4).

**Table 4:** Comparison of awareness rate of physical and mental health between two groups of patients before and after intervention

Index		Awareness rate of physical and mental health (%)		χ ^2^	*P*
		Yes	No		
Baseline	Intervention	36.7	63.3	0.352	0.553
	Control	33.3	66.7		
Final line	Intervention	57.7	42.3	5.781	0.016
	Control	41.4	58.6		

### Harms/adverse events

There were no other serious or mild adverse events.

## Discussion

In this manuscript, the main outcome indicators and secondary outcome indicators of the RCT were collected strictly according to the experimental scheme, consistent with the protocol.

In our study, we found that a 10-week mindfulness-based therapy intervention significantly improved depression. Concurrently, the improvement in systolic blood pressure may be attributed to the relaxation of an overactive sympathetic nervous system caused by long-term stress. Additionally, while there was initially a minor reduction in anxiety levels, this reduction became significant by the end of the 10-week intervention. These findings suggest that mindfulness-based therapy has a positive effect on both depression and anxiety, with a particular emphasis on depression. It's noteworthy that hypertension often coexists with anxiety and/or depression. Our data further supports a proactive approach to mindfulness-based intervention, particularly in the early stages, for hypertensive patients predisposed to anxiety or depression, to minimize or reduce disease progression.

Effective management of blood pressure (BP) can reduce the incidence of depression [35], anxiety, and BP fluctuations. Furthermore, exercise therapy included in mindfulness therapy can lower blood pressure, primarily by helping participants adopt healthy lifestyle habits, as demonstrated in Oliveira's study [36]. Therefore, intervention for depression and/or anxiety is an important and effective auxiliary in managing hypertensive patients with depression and/or anxiety. Our aim was to effectively manage BP in these stressed and depressed patients. Our current study demonstrated that mindfulness therapy improved the level of depression in the patients. We observed a significant reduction in systolic BP with mindfulness-based therapy, in addition to reductions in depression and anxiety, compared to the conventional approach among patients in the community. Our findings are consistent with previous studies showing that mindfulness-based therapy can reduce hypertension [20] or stress levels among in-hospital patients [35]. Interestingly, we observed no significant difference in diastolic BP between the mindfulness-based therapy group and the control group.

We recognize that stress and generalized anxiety disorder are not always identical or directly related. However, there are linkages between stress and generalized anxiety disorder, with chronic stress contributing to the relapse of generalized anxiety disorder [37]. This observation provides some explanation for our current finding, which suggests that mindfulness intervention appears effective in managing hypertension, as well as reducing anxiety and depression levels, despite the fact that stress was not the primary focus of the study [21]. Our data suggests that mindfulness-based therapy is more effective than simple health education in managing systolic BP and psychological stress among hypertensive patients. It is well known that systolic BP is closely related to both physical and psychological status [38], particularly in immediate responses.

Thus, mindfulness-based therapy may help soothe the minds of participants with high stress levels and sympathetic signaling via noradrenaline [39]. This speculation is supported by Strandberg and Pitkala, who found positive intervention outcomes for hypertension participants with reduced depression [40]. In our study, we did not observe a significant reduction in diastolic BP following mindfulness-based therapy compared to the control. Diastolic BP, reflecting the elasticity of arteries, is not easily or rapidly modified, even with strong medication [41]. However, while there may not be a significant effect of mindfulness-based therapy on diastolic BP, we did observe an improving trend compared to that of the healthy educational intervention alone. Conversely, systolic BP is partially regulated by environmental temperature [42]. Seasonal variations, with our study starting in the summer and drawing comparisons with data collected in winter, may have had a partial impact on BP measurements [43]. Our participants were recruited in early autumn (September) and completed the study by winter (February), suggesting seasonal dependence. We will investigate seasonal interference in future studies.

Our data demonstrated that the self-efficacy score and mental health state of participants in the mindfulness-based group were significantly higher than those in the control group after intervention. Affected by hypertension and poor mental state, participants experience varying degrees of negative psychological emotions, such as anxiety and depression. These negative emotions not only impact the patient’s compliance with treatment but also exacerbate mental stress, further contributing to mental deterioration, creating a dangerous cycle [44]. Mindfulness-based therapy guides patients to manage themselves through mindfulness meditation, helping them to observe the generation and dissolution of their own emotions. This intervention method significantly improves the patient’s attention [45] through long-term mindfulness meditation training. Consequently, the intervention enhances the patient's mindfulness level, thereby further improving the participant’s mental health in a positive feedback loop [46].

The awareness rate mainly refers to the cognition or opinion of an objective situation or fact, especially regarding various health issues that require raising public awareness [47]. There was a significant difference in the awareness rate between the intervention and control groups, further supporting the effectiveness of the intervention for managing patients with comorbid hypertension and anxiety/generalized anxiety disorders. Our current findings are consistent with others, showing that mindfulness intervention appears to be effective in managing hypertension, as well as reducing anxiety and depression levels, although stress was not the focus of the current study [21]. Our study demonstrated that after completing the dual intervention of mindfulness therapy and health education, the awareness rate of physical and mental health among participants in the intervention group increased significantly. Consequently, participants are better equipped to deal with blood pressure changes effectively, in addition to experiencing improvements in physical and mental health.

Following the intent-to-treat analysis, the statistical power of the trial has been maintained, despite the relatively low number of patients who did not progress to the final follow-up. The results showed that mindfulness therapy has a beneficial effect following clinical application. We observed that one participant in the intervention group, who was in a state of mild depression but did not complete all treatment sessions, experienced a significant decrease in depression score and improved mental health. Conversely, Ma et al*.* adhered to medication and maintained good mental health [48]. In the control group, two participants who completed all hypertension health education sessions experienced significantly decreased depression scores and improved mental health. Our data suggest that scientific and health education can improve the mental health of hypertension patients with depression/anxiety. However, a small number of patients did not achieve significant improvement, which warrants further study.

We would like to emphasize that hypertensive patients combined with depression and anxiety have a strong impact on their personal lives, in addition to incurring significant costs for medical attention and social support. Mindfulness therapy reduces financial costs as well as adverse effects associated with medical intervention. Furthermore, group psychotherapy (mindful therapy) has brought convenience with the development of networks. Among patients with poor responses to drug treatment, the combined application of mindfulness-based cognitive therapy can serve as a promotional model to alleviate symptoms of depression and anxiety in hypertensive patients.

There are several limitations in the present study. The random numbers generated were kept in opaque envelopes, which were blinded to both the individuals organizing and analyzing the data. The intervention group was assigned to the West Campus of Gem Flower Hospital, while the control group was assigned to the East Campus. Although this approach aimed to minimize interference between the intervention and control individuals, it remains a limitation in the double-blind RCT, which will be further investigated in the future.

Originally, there were 60 participants; however, several participants were withdrawn from either the intervention (four) or the control group (one) due to physical reasons or antidepressant medication. The study started in September (autumn) and was completed in early February (winter) of 2020, which could have interfered with blood pressure (BP) and depression/anxiety scores. BP and depression/anxiety scores may have been affected by the following reasons: Firstly, people typically engage in fewer outdoor activities and have fewer social connections in the winter [49], which can exacerbate anxiety and depression caused by home isolation. This could lead to bias in the detected outcomes because isolation can contribute to depression or anxiety [50], resulting in deteriorated mental health.

Secondly, outcomes might be affected due to the impact of COVID-19. Measures and public awareness campaigns about COVID-19 were stricter and more frequent in winter [51, 52], which could increase people's psychological pressure. The participants were from one community; however, future studies will be conducted across multiple community health centres. Additionally, our registry protocol was retrospective; however, during program implementation, we strictly followed the outcome measures and reported them. In future research, we will conduct multicentre, large-sample intervention studies to provide more clinical evidence for mindfulness-based interventions.

## Conclusion

Mindfulness-based, short-term focused interventions provide modest relief for depression and/or anxiety and are effective in lowering blood pressure and improving self-efficacy scores.

## Supplementary Information

**Supplementary Material 1.**

## Data Availability

The original contributions presented in the study are included in the article/supplementary material. Further inquiries can be directed to the corresponding authors.
